# Overactive bladder

**DOI:** 10.12688/f1000research.7131.1

**Published:** 2015-12-07

**Authors:** Karen M. Wallace, Marcus J. Drake

**Affiliations:** 1 Bristol Urological Institute, Southmead Hospital, Bristol, UK

**Keywords:** Overactive bladder syndrome, urinary urgency, nocturia, detrusor overactivity, mirabegron

## Abstract

Overactive bladder syndrome is highly prevalent, and increasingly so with aging. It is characterized by the presence of urinary urgency, and can be associated with incontinence, increased voiding frequency, and nocturia. Assessment needs to exclude serious medical disorders that might present with similar symptoms, and a bladder diary is an invaluable part of understanding the presentation. Initial management is conservative, comprising education, bladder training, and advice on fluid intake. Drug therapy options include antimuscarinic medications and beta-3 adrenergic receptor agonists. Persistent overactive bladder syndrome, despite initial therapy, requires a review of the patient’s understanding of conservative management and compliance, and adjustment of medications. For refractory cases, specialist review and urodynamic testing should be considered; this may identify detrusor overactivity or increased filling sensation, and needs to exclude additional factors, such as stress incontinence and voiding dysfunction. Botulinum neurotoxin-A bladder injections can be used in severe overactivity, provided the patient is able and willing to do intermittent self-catheterisation, which is necessary in about 5% of treated patients. Sacral nerve stimulation and tibial nerve stimulation are other approaches. Major reconstructive surgery, such as augmentation cystoplasty, is rarely undertaken in modern practice but remains a possibility in extreme cases.

## Introduction

The International Continence Society ^1^, with slight modification by the International Consultation on Incontinence Research Society ^2^, states that overactive bladder syndrome is urinary urgency, with or without urgency incontinence, usually with increased daytime frequency and nocturia, if there is no proven infection or obvious pathology.

The components of the symptom syndrome of overactive bladder syndrome are as follows. The key symptom is urinary urgency, which is the complaint of a sudden compelling desire to void that is difficult to defer ^1^. Urgency should not be confused with a strong desire to void, which is effectively the normal sensation when functional bladder capacity is reached. Patients often express anxiety around the “fear of leakage” due to urgency. It is the “fear of leakage” and the “fear of pain” that differentiates patients suffering from urgency in overactive bladder syndrome from those with bladder pain syndrome ^3^. In overactive bladder syndrome, urgency is typically felt at the perineum or the base of penis or vagina/urethra. In contrast, in bladder pain syndrome suprapubic pain is characteristic, though additional perineal (urethral/vaginal/penile) discomfort can occur ^4^.

Urgency urinary incontinence is defined as involuntary leakage of urine, accompanied or immediately preceded by urgency ^1^. Urgency urinary incontinence can cause concern to the patient, such as social or hygiene effects, but the extent to which patients complain of such problems is very varied.

Increased daytime frequency is the complaint made by the patient who considers that he or she voids too often by day ^1^. This standardised definition does not include any minimum number of voids, since there is considerable overlap between normal and overactive bladder syndrome in terms of objective voiding frequency. Currently, evidence to set a threshold for defining increased daytime frequency is lacking, so a minimum number of voids is not included in the definition.

Nocturia is the complaint made by the individual who has to wake at night one or more times to void ^1, 5^.

Mixed urinary incontinence encompasses overactive bladder syndrome and stress urinary incontinence. The complaint is of involuntary leakage associated with urgency and with exertion, effort, sneezing, or coughing ^1^.

The current definition of overactive bladder syndrome is a symptomatic diagnosis. In contrast, detrusor overactivity (DO) is a urodynamic observation, characterised by involuntary detrusor contractions during the filling phase, which may be spontaneous or provoked ^1^. These are not terms that can be interchanged, as overactive bladder syndrome sufferers may not have detrusor activity on urodynamic testing.

The overall prevalence of overactive bladder syndrome is approximately 12% ^6^. Men have a higher prevalence of “overactive bladder syndrome dry”, meaning urgency without urgency urinary incontinence, and women had a higher prevalence of “overactive bladder syndrome wet” ^7^. The differing prevalence of incontinence is presumed to be due to the relative weakness of the bladder neck and urethral sphincter mechanism in women. The prevalence of overactive bladder syndrome wet in women was also noted to increase significantly with age, from 2.0% in those aged between 18 and 24 to 19.1% in those 65 to 74 years of age.

Overactive bladder syndrome affects many patients and their quality of life; the symptom of urgency having the biggest impact. A large study carried out in the US, UK and Sweden reported that storage symptoms held greater impact than other lower urinary tract symptoms ^8^. Substantial cost is associated with overactive bladder syndrome. The population cost estimates are dependent on the accuracy of prevalence data. In estimate, the average annual per capita cost of overactive bladder syndrome in the US was $1925 in 2000, bringing a national cost of tens of billions of dollars ^9^. This can be inferred to include indirect impact, such as falls when having to void urgently ^10^, and impaired social or occupation functioning ^11^.

## Pathophysiology and aetiology

As overactive bladder syndrome is a condition based on symptoms, there is no equivalent animal model ^12^. Instead, surrogates are used, and assumptions have to be made, which should not be over-interpreted. The research focus has narrowed in on three key aspects.

The first of these is lower urinary tract sensory activity, which is due to the sensations driving the symptom syndrome. Sensory activity involves the understanding of afferent signalling, including signal transduction and afferent traffic, gating, sensitization, and conscious perception. The lower urinary tract nerve endings are densely concentrated under the urothelium. In this location, they may be exposed to release of mediators by the urothelium ^13, 14^, cellular influences, and cytokines ^15^. Thus, many patients may experience urinary urgency as a consequence of altered sensory input.

The second aspect is motor control, which is due to the presence of detrusor overactivity in many people with overactive bladder syndrome. Motor function involves the processes giving rise to the contractility in the detrusor muscle of the bladder. Many changes in properties of bladder smooth muscle have been described in overactive bladder syndrome and detrusor activity, leading to the “myogenic hypothesis of detrusor activity”. This suggests that overactive detrusor contractions result from increased excitability and spread of contraction within the muscle ^16^. This may be further altered *via* the efferent nerves, interstitial cells and local mediators ^17^. Such excitation is normal during voiding, but should not occur during storage, since there are CNS inhibitory influences to suppress them.

The third aspect of research focus is on reflexes of the lower urinary tract, since bladder urine storage requires inhibition of the muscle ^18^, such that disinhibition of bladder motility or voiding can be presumed to cause detrusor overactivity or urgency urinary incontinence. The motor behaviour of lower urinary tract reflexes is underpinned by the ascending afferents of sensory information that are integrated at various CNS levels (notably the brainstem and the sacral spinal cord) ^19, 20^. The potential importance of the reflexes is encapsulated in the neurogenic hypothesis of detrusor overactivity, which states that detrusor overactivity arises from generalized, nerve-mediated, excitation of the detrusor muscle ^21^.

Overactive bladder syndrome prevalence has some gender disparity, and there is a higher prevalence in women, particularly in younger people. A small influence of racial factors is also present, with slightly higher prevalence among African Americans than among Hispanics and whites, for both men and women ^22^.

Neurological disease is highly associated with lower urinary tract dysfunction, due to impaired regulatory influence of the innervation on the lower urinary tract. The CNS should allow people to store urine asymptomatically; consequently, neurological disease commonly impairs urine storage, leading to detrusor overactivity.

Bladder outlet obstruction has also been considered to play a part in detrusor overactivity and overactive bladder syndrome, although the relationship is not clear. Detrusor overactivity and overactive bladder syndrome increase with aging, and co-existing bladder outlet obstruction may enhance this effect.

Overactive bladder syndrome can often be described by patients also reporting functional problems, including altered bowel function ^23^ and fibromyalgia ^24^.

## Clinical assessment

Given that potentially serious mechanisms could present with similar features, assessment needs to exclude disorders such as neurological, malignant, or systemic disease. Careful history taking, physical exam and urinalysis are mandatory ^25, 26^. The history should include symptoms such as urgency, urgency incontinence, nocturia, increased frequency, dysuria, haematuria, and lower urinary tract pain. These symptoms are often best explored with a symptom-based questionnaire. The clinician should also enquire about fluid intake to assess for polydipsia and stimulants that could worsen lower urinary tract symptoms ^27^. Other symptoms considered may include new-onset tremor or erectile dysfunction, which may hint at neurological disease. Other existing medical conditions, such as closed angle glaucoma, history of urinary retention, and cognitive impairment, should be ascertained, as these are relative contraindications to anti-muscarinic therapy. Likewise, hypertension may be a contraindication to beta-3 adrenergic agonist therapy.

A general examination, abdominal and pelvic examination, and a basic neurological examination should be carried out. By palpating the abdomen after recent voiding it is possible to identify significant post void bladder residual, noting that such a situation may impair response to bladder storage medications. Urinalysis is crucial in all assessments to exclude urinary tract infections, leukocyturia, and haematuria.

Evaluating frequency of voiding and nocturia is carried out by getting the patient to complete a frequency volume chart or bladder diary. In overactive bladder syndrome, the pattern of voided volumes is often characteristically erratic. On a frequency volume chart, frequency is the number of voids recorded during waking hours, including the last void before sleep and the first void on waking and rising in the morning ^1^. Completion of such a record can identify hindering factors that affect management, such as nocturnal polyuria, or potential factors leading to sleep disturbance ^28^. The maximum voided volume recorded can indicate the severity of overactive bladder syndrome and detrusor overactivity. A bladder diary is used to capture information beyond that seen on a frequency volume chart, such as symptom scores. Good examples of scores used in a diary include the ICIQ Bladder diary score ^29^ or the Patient Perception of Intensity of Urgency Scale (PPIUS) ^30^. Scales allow patients to rank a score for each time they void, such as 0 (no urgency) through to 4 (urgency incontinence: leaking before arriving at the toilet). A quantitative method of assessing the bladder diary is the Total Urgency and Frequency Score (TUFS), where the daily sum of the PPIUS scores is calculated ^31^.

Urodynamics, cystoscopy, and diagnostic urinary tract ultrasound should not generally be used in initial workup of the uncomplicated patient ^25, 26^. They may have a role where symptoms persist despite compliance with appropriate initial therapy.

## Treatment

### Initial treatment outline

The nature of the overactive bladder syndrome definition enables an empirical diagnosis, allowing clinicians to initiate preliminary treatment. Thus, it is common practice to employ conservative management and oral pharmacotherapy without a urodynamic diagnosis. In overactive bladder syndrome, management of expectations necessitates taking an honest approach explaining the nature of the symptom syndrome and that “cure” is not a realistic goal. Lifestyle interventions play a large part in managing overactive bladder syndrome: educating the patient about their condition and certain aspects that influence it, such as volume of fluid intake, smoking cessation, and making certain dietary alterations. Bladder training and pelvic floor muscle training are valuable components ^32^ and may re-establish some inhibitory control over bladder storage.

Drug treatments should be initiated after conservative methods have been tried. Anti-muscarinic medication is a main drug class used for treatment ^33^. The efficacy of the drugs is counterbalanced by potential side effects that the patient should be warned about, such as dry mouth, constipation, cognitive effects, and visual impairment, amongst others. The cognitive effects may prevent the use of antimuscarinics in older patients ^34^. Accordingly, a balance of beneficial and adverse effects has to be considered when prescribing therapy and reviewing outcomes ^35^. As a drug class, persistence rates are low (12–39% at 12 months and 6–12% at 24 months) ^36^. Part of this may result from the well-recognised placebo effect in overactive bladder syndrome drug therapy ^37^, which may bring an initial but poorly sustained response, and may reflect the potentially heterogeneous populations included within the clinical trials.

A beta-3 adrenergic agonist has also been introduced as a means of medical management of overactive bladder syndrome, which works by detrusor relaxation through binding to the subtype sympathetic receptor. Large phase 3 clinical trials have found therapeutic efficacy ^38, 39^ and a differing adverse effect profile from antimuscarinics. Accordingly, mirabegron can be considered for older patients ^40^. Mirabegron can bring symptom improvement to people who have not had adequate response to antimuscarinics ^41^. Combination therapy, using both antimuscarinic and mirabegron, is under development ^42, 43^.

Men with overactive bladder syndrome can legitimately be prescribed antimuscarinic drugs ^44^. Men with concurrent voiding symptoms should be given an additional alpha-1 adrenergic blocker as first line ^45^. While caution is advised in prescribing antimuscarinics to men ^45^, long-term (up to a year) combination therapy of alpha blocker and antimuscarinic in men with moderate-to-severe lower urinary tract symptoms is associated with a very low rate of acute urinary retention ^46^.

The treatment will need to be followed up and tailored according to the efficacy and adverse effects of the drugs ^45^. Once medication has begun, repeating a symptom assessment questionnaire can be used to evaluate response.

### Specialised evaluation and management outline

If the initial treatment seems inadequate, then a specialist referral is appropriate. In this situation it is necessary to review the diagnosis, look for complicating factors, ensure that suitable initial therapy has been given and complied with, and that response has been appropriately evaluated. Adverse effects can influence willingness to continue medication ^47^.

Urodynamic evaluation is potentially part of specialist assessment. It applies when conservative and medical management is suboptimal and if overactive bladder syndrome is having a significant impact on a patient’s quality of life. The patient should be sufficiently healthy and in a position to consider more invasive therapeutic intervention if appropriate. The aim of urodynamics is to recreate the patient’s symptoms and identify what factors can influence the treatment. The diagnoses associated with overactive bladder syndrome in this investigation are detrusor overactivity and increased filling sensation. Detrusor overactivity can be phasic (characterised by increasing amplitude in contractions when the bladder volume increases) or terminal (a single involuntary detrusor contraction at cystometric capacity, causing incontinence often leading to bladder emptying). It is important to realise that some patients with detrusor overactivity are asymptomatic and do not, strictly-speaking, have overactive bladder syndrome. In women, detrusor overactivity may not be present in some patients with overactive bladder syndrome ^48, 49^. If detrusor overactivity is absent, the patient may report increased bladder sensation during urodynamics, that is, an early and persistent desire to void ^1^. If detrusor overactivity isn’t observed during bladder filling, then a provocation test can be initiated, such as generating the sound of running water. If urodynamic stress incontinence is seen, it should be ascertained whether the urodynamic stress incontinence leads to a sensation of urgency, which can occur due to urethral stimulation, and should not be confused with detrusor overactivity. The voiding phase in a urodynamic test may reveal bladder outlet obstruction or incomplete bladder emptying.

### Specialist treatment outline

Conservative methods are revisited, including bladder training, pelvic floor muscle training, and fluid advice, as this important aspect is often overlooked in initial treatment, and specialist centres may be better set up for more effective delivery. Likewise, pharmacotherapy is fully reviewed, based on antimuscarinics and/or beta-3 adrenergic agonist, to ensure a suitable agent, dose level, and timing.

Interventional therapy may be offered, potentially including the following options.

Intravesical botulinum neurotoxin-A injection has become established as a mainstream method of management of refractory overactive bladder syndrome ^26^. The technique involves injection of 100 units of onabotulinum-A throughout the bladder wall ^50– 52^. Prior to injection, patients need to be taught intermittent self-catheterisation, as there is a small risk of significant urinary retention after injections. The response is temporary, but the majority of patients will respond to repeat injections, which typically are needed between six months and a year later. The major adverse effect is urinary tract infection. Furthermore, the extent of symptom control fluctuates, peaking at about a month, and declining thereafter until retreatment.

Sacral nerve stimulation, can be used as an alternative to botulinum injections, and patients who are dissatisfied with botulinum toxin-A treatment, or in whom such treatment fails, can respond successfully to sacral neuromodulation ^53^. Sacral nerve stimulation follows a test phase, in which temporary electrodes are placed adjacent to the S3 sacral nerve root and connected to an external battery pack. If sufficient symptomatic improvement is seen, the definitive electrode can be placed, with a subcutaneous stimulator pack. The implant is expensive, and the battery pack has to be replaced once it has discharged. However, if viewed in the longer term, the cost may be comparable to that of other treatments ^54^. Incontinence episodes and voiding frequency are both reduced while receiving sacral nerve stimulation, and this is associated with improved quality of life ^55, 56^. Device-related adverse events were reported in one study for 16% of subjects during test stimulation and 30% of subjects post-implant ^55^.

Tibial nerve stimulation is achieved with a fine needle electrode temporarily placed at the level of the medial malleolus of the ankle, observing for intrinsic foot muscle contraction as an indicator of proximity to the nerve ^57^. The stimulus is applied at close to tolerance threshold for half an hour. Treatment is repeated at weekly intervals for an induction phase, and then the interval is increased to maintain efficacy. This approach is labour-intensive, though some patients can learn to self-administer. Efficacy is comparable to that seen with antimuscarinics, but with fewer adverse effects ^58^. Due to the intensive nature of treatment delivery and uncertain long-term efficacy, tibial nerve stimulation is not widely used in mainstream healthcare.

The exact pathway for interventional therapy options varies between centres. In addition, patient preferences are an important factor when considering next steps for managing overactive bladder syndrome persisting despite drug therapy ^59^.

Augmentation cystoplasty is a rare procedure in modern management of overactive bladder syndrome ^60, 61^. It requires splitting the bladder in half, and incorporating an isolated section of small intestine that has been detubularised. The operation is a major undertaking, and there is a risk of major complications, such as rupture of the reconstructed bladder, metabolic effects (due to reabsorption of toxins from the urine by the bowel segment), new bowel symptoms, and sepsis. Accordingly, this option is only considered once all alternatives have been fully explored.

## Conclusions

Overactive bladder syndrome is a common problem, with potentially substantial impact on quality of life. Assessment requires exclusion of serious diagnoses and other influential factors. Management is initially conservative, followed by a tailored use of antimuscarinics or mirabegron, with follow up review. Refractory cases need specialist reassessment, typically with urodynamic testing. Management may then require botulinum neurotoxin injections, sacral nerve stimulation, tibial nerve stimulation, or even augmentation cystoplasty. Each option has significant considerations, which must be fully evaluated with the patient to avoid major problems.
